# Supplementary figures and images for: Evaluation of the validity of the Psychology Experiment Building Language tests of vigilance, auditory memory, and decision making
Source: PeerJ. 2016 Mar 15;4:e1772. doi: 10.7717/peerj.1772 (PMC4806597; doi:10.7717/peerj.1772)

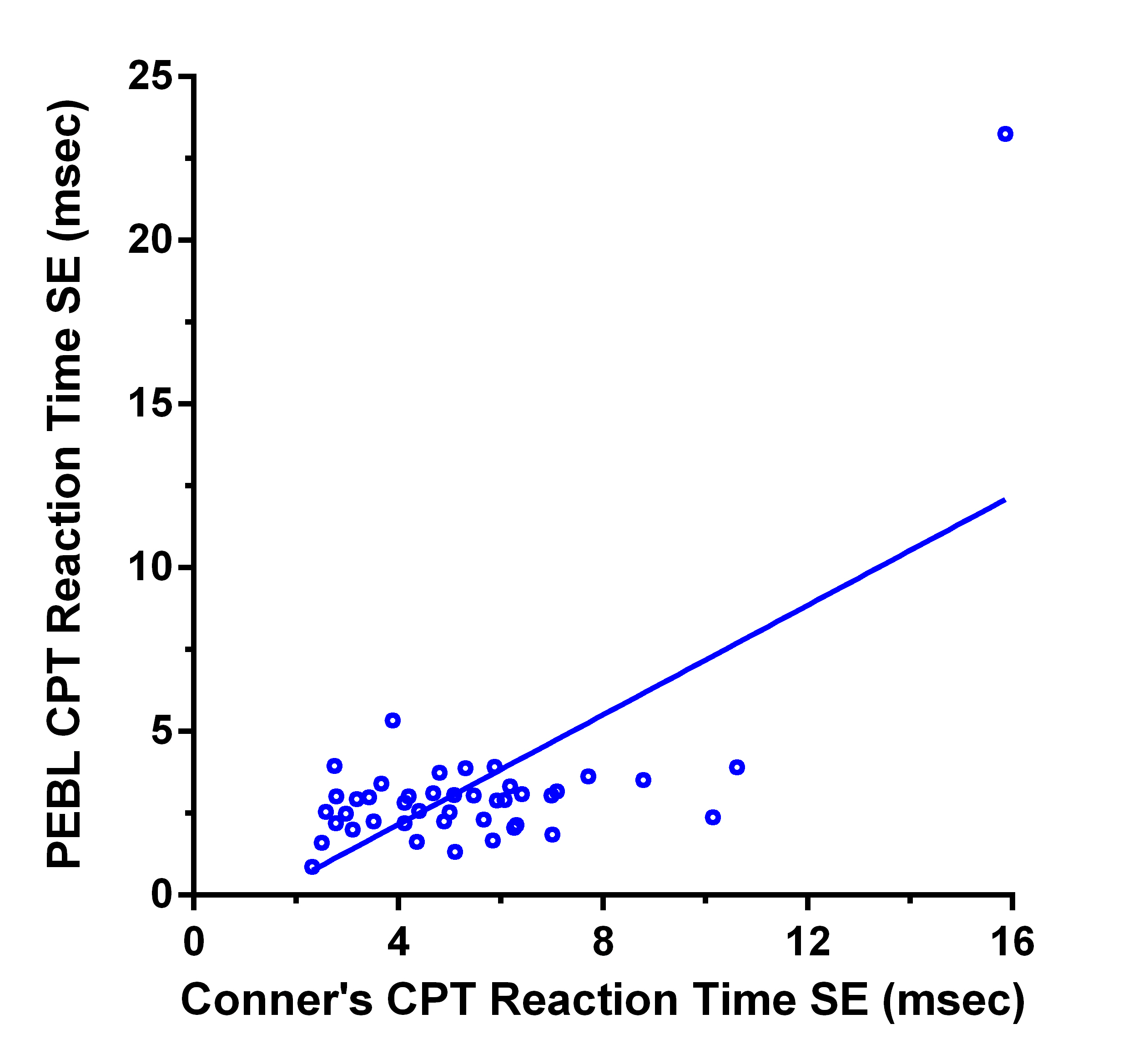

Supplement: Figure S1 — Removal of one extreme score (upper-right) reduced the proportion of variation accounted considerably (R2 = .44, p < .0005 to R2 = .04, p = .19). [file peerj-04-1772-s003.jpg]
